# Secondary Hemophagocytic Lymphohistiocytosis in an Infant with Wolman Disease

**DOI:** 10.4274/tjh.2015.0454

**Published:** 2017-08-02

**Authors:** Aynur Küçükçongar Yavaş, Betül Orhaner, Pınar Genç, Nevin Kılıç, Hakan Erdoğan, Özlem Özdemir, Arzu Ekici

**Affiliations:** 1 Şevket Yılmaz Training and Research Hospital, Clinic of Pediatric Metabolism, Bursa, Turkey; 2 Şevket Yılmaz Training and Research Hospital, Clinic of Pediatric Hematology, Bursa, Turkey; 3 Şevket Yılmaz Training and Research Hospital, Clinic of Pediatrics, Bursa, Turkey; 4 Şevket Yılmaz Training and Research Hospital, Clinic of Pediatric Nephrology, Bursa, Turkey; 5 Şevket Yılmaz Training and Research Hospital, Clinic of Pediatric Neurology, Bursa, Turkey

**Keywords:** Wolman disease, Hemophagocytic lymphohistiocytosis, hemophagocytosis

A 2-month-old girl presented with vomiting, fever, failure to thrive, and diarrhea. She was born to consanguineous parents. She was irritable and pale and she had hepatosplenomegaly (Figure 1). Her weight and height were below the 3^rd^ percentile. Initial hemoglobin count was 7.6 g/dL, white blood cell count was 12x10^9^/L, platelet count was 92x10^9^/L, triglyceride level was 361 mg/dL (reference range: 40-150 mg/dL), and ferritin level was >1650 ng/mL. According to bone marrow aspiration, numerous examples of hemophagocytosis were observed (Figure 2). She was diagnosed with hemophagocytic lymphohistiocytosis (HLH) because of prolonged fever, organomegaly, bicytopenia, high levels of ferritin, and bone marrow findings. Enzymatic analyses were performed for lipid storage disorders. The lysosomal acid lipase (LAL) activity was <0.02 nmol/punch/h (reference range: 0.07-2.3 nmol/punch/h). Based on reduced LAL activity, she was diagnosed with Wolman disease. Although treatment and supportive therapy were applied, the patient died 1 month later.

Exon 4 heterozygous variation was found at the LIPA gene location c:260G>T (GGC>GTC), p.Gly87Val. Sequence analysis of all coding regions of the LIPA gene presented heterozygote NM_000235(LIPA_vENST00000336233):c.260G>T(p.Gly87Val) variation in both parents. This variation was reported as a disease-causing variant by Pagani et al. [1].

It was discussed in cases of Wolman disease that the pathophysiological role of cholesteryl ester induces inflammasome activation in macrophages, leading to secondary HLH [2].

## Figures and Tables

**Figure 1:**
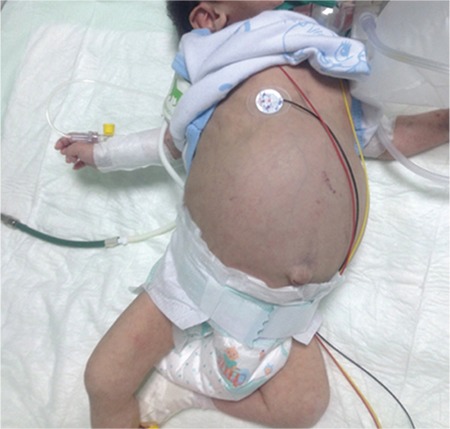
Two-month-old female patient with Wolman disease, showing abdominal distension.

**Figure 2:**
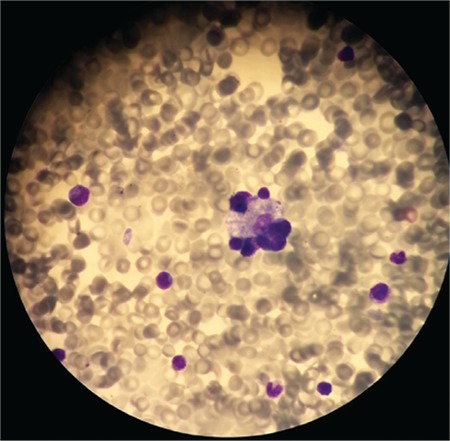
Hemophagocytosis in the bone marrow of our patient.
